# Cumbersome but desirable—Breaking the code of everyday cycling

**DOI:** 10.1371/journal.pone.0239127

**Published:** 2020-09-14

**Authors:** Helga Birgit Bjørnarå, Thomas Westergren, Liv Fegran, Saskia J. te Velde, Aslak Fyhri, Benedicte Deforche, Lars Bo Andersen, Sveinung Berntsen, Elling Bere

**Affiliations:** 1 Faculty of Health and Sport Sciences, University of Agder, Kristiansand, Norway; 2 Department of Safety and the Environment, Institute of Transport Economics, Oslo, Norway; 3 Department of Public Health and Primary Care, Faculty of Medicine and Health Sciences, Ghent University, Ghent, Belgium; 4 Physical Activity, Nutrition and Health Research Unit, Faculty of Physical Education and Physical Therapy, Vrije Universiteit Brussel, Brussels, Belgium; 5 Western Norwegian University of Applied Sciences, Faculty of Education, Arts and Sport, Sogndal, Norway; 6 Department of Health and Inequalities & Centre for Evaluation of Public Health Measures, Norwegian Institute of Public Health, Oslo, Norway; University of Sheffield, UNITED KINGDOM

## Abstract

**Introduction:**

Cycling for transport could integrate physical activity (PA) into daily routines and potentially increase total PA levels. However, for parents with young children, most factors affecting transport mode choice tend to facilitate car use. Greater insight is necessary into reasons for (not) using sustainable transport modes in parents with young children. Therefore, the objective of this study was to explore the experiences, including motives, perceptions, attitudes, and norms, of parents of young children by using an e-bike, a longtail bike, and a traditional bike for everyday travel to the workplace, kindergarten, and the grocery store during the autumn, winter, and spring, in nine months.

**Methods:**

Semistructured focus group interviews were conducted with 18 parents of young children residing in southern Norway. Parents were recruited through Facebook announcements and direct contact with kindergartens, selected organisations, and companies in the Kristiansand municipality. Data were analysed by systematic text condensation by using NVivo V.11.

**Results:**

Participants’ experiences were summarised by three main themes: ‘cycling is cumbersome’, ‘cycling reflects the desirable me’, and ‘breaking the cycling code’. Time use, planning, logistics, wet and cold weather, long distances, and no cycling habit were frequently mentioned barriers, and the most notable facilitator was the children’s attitude towards cycling. In general, children loved to cycle and preferred cycling to driving. Additionally, the freedom and independence of cycling were emphasised and valued.

**Conclusion:**

In challenging weather conditions, parents of young children may experience cycling as cumbersome but desirable, and bike access could increase the feasibility of daily cycling.

## Introduction

The lack of time [1] and stress [2] have been negatively associated with physical activity (PA) in adults. Cycling for transport could integrate PA into daily routines, potentially increasing total PA levels [3, 4] in a time-efficient manner. However, for parents with young children, factors such as both parents being employed, organised leisure activities (i.e. of older siblings), perceived time pressure, and enhanced car access tend to facilitate car use [5]. Modern family life in the Nordic countries relies on a norm of involved parenthood [6], entailing difficulties in balancing work and life, hobbies, and social networking [7]. Time and childcare management in dual-earner families include strategies of delegating childcare to kindergartens, relatives, and information technology; alternating the routine of care between the two parents; and multitasking to increase time-efficiency and be involved with the children [6]. Further, some periods of the day are perceived as more time-pressured than others, for example, the morning routine, entailing a feeling that time just flies and nothing is being accomplished [8]. Nevertheless, varied travel patterns emerge among families with young children, and it has been proposed that the cohort of millennials, now approaching parenthood, might be more open to sustainable alternatives to car use and car ownership than earlier generations were [5]. Considering that a moderate tracking of PA during early childhood [9] and from childhood to adulthood [10] has been suggested, healthy lifestyle behaviours such as PA, including active travel, should be promoted early in life [9]. Being transported to kindergarten by bike instead of by car could familiarise children with alternative modes of transport, which may establish healthy and sustainable transport and PA habits. Thus, parental transport mode choices could have a positive influence on both their own and their children’s current and future health.

Electric-assisted bicycles (e-bikes) enable the maintenance of speed with less effort, reducing common barriers to traditional pedal cycling such as poor fitness, hilly terrain, long distances, a lack of time, and a lack of end of trip facilities (e.g. changing rooms and showers) [11], while attaining the recommended amount of moderate-to-vigorous intensity physical activity (MVPA) [12]. Notably, one limitation of both traditional bikes and standard e-bikes is restricted carrying capacity [13], which applies to a lesser extent to longtail bikes and other types of cargo bikes constructed for transporting equipment and children. Where the environment is favourable, active travel modes are feasible for families with young children. In Copenhagen, approximately 25% of families with at least two children possess a cargo bike [5], and under favourable conditions and circumstances, mothers in United States with access to a cargo bike consider it a realistic mode of choice and a substitute for a car [14]. Likewise, parents using e-bikes, including cargo e-bikes, for everyday travel in the San Francisco Bay Area, emphasise advantages such as greater freedom of travel and parking compared with a car [15]. Additionally, cyclists are consistently found to be the happiest commuters, contentment for which four components were recently proposed: commuting control and ‘arrival-time reliability’, the mental effects of MVPA on improving one’s mood, sensory stimulation reaching enjoyable levels, and social interactions are more likely to occur [16].

However, greater insight is necessary into reasons for using or avoiding sustainable transport modes in parents with young children [5]. We recently showed in the intervention study ‘From cars to bikes’ (CARTOBIKE) that Norwegian parents of young children increased cycling for transport and decreased car use when provided free access to different bike types for nine months [17]. Few studies have assessed the feasibility of or parents’ experiences with everyday cycling; however, a US study reported that using a cargo e-bike for family transport was far more time reliable during rush hour than driving a car, and the greater mobility and freedom of travel provided by the cargo e-bike were highly valued [15]. Additionally, an intrinsic utility related to the commute by bike has been described by adult e-bike users in the Netherlands, entailing a feeling of well-being from being physically active outdoors, combined with an allowance to prepare mentally for the day ahead, or to disconnect from work on the way home in the afternoon [18].

Qualitative research methods are suitable for the in-depth exploration of selected topics, such as the feasibility of cycling for transport, and offer a functional tool for an increased understanding of the complexities of travel behaviour [19]. More specifically, focus groups can be a valuable research tool to explore not only experiences but also interactions with a topic, offering a compromise between the strengths of participant observation and those of individual interviews [20]. Participant dynamics enables the exchange and comparison of experiences, and potential disagreements could result in more complete descriptions than would have been obtained from individual interviews [21]. According to Morgan [20], focus groups are suitable both for exploring experiences and opinions, providing insights into complex behaviours and motivations. Thus, the information gathered from focus groups could inform further interventions to promote everyday cycling in parents of young children or intervention evaluation. Therefore, the objective of this study was to explore the experiences, including motives, perceptions, attitudes, and norms, of parents of young children by using different bike types (S1 Fig) for everyday travel to the workplace, kindergarten, and grocery store during the autumn, winter, and spring, by using semistructured focus group interviews.

## Methods

### Study design and sample

This qualitative study was part of the research project CARTOBIKE (described in detail elsewhere) [17, 22]. A convenience sample was recruited for the project, comprising 36 parents with children attending kindergarten, residing in southern Norway. Inclusion criteria were (i) being able to read and understand Norwegian, (ii) having one child born in year 2013, 2014, or 2015 attending kindergarten, (iii) being responsible for bringing/picking up the child in the kindergarten ≥5 times per week, or at least half of the times, (iv) residing 2–10 km from the workplace, (v) residing <3 km from the kindergarten and the grocery store, (vi) having car access, (vii) being between 167 and 190 cm tall (due to the size of accessible bikes), and (viii) having the ability to store the bikes indoors. Further exclusion criteria were (i) being physically active, namely fulfilling the PA recommendations; (ii) having cycled more than once weekly throughout the last 12 months to the workplace, kindergarten, or the grocery store; and (iii) suffering from severe cardiovascular diseases or upper respiratory tract diseases. Participants in the intervention group (*n* = 18) completed the following intervention arms in random order from September 2017 through May 2018: (i) three months’ access to an e-bike with a trailer (*n* = 6); (ii) three months’ access to a human-powered longtail cargo bike (*n* = 6); and (iii) three months’ access to a traditional bike with a trailer (*n* = 6) in the autumn (September–November), winter (December–February), and spring (March–May) season, respectively. Detailed information on the bikes used in the project and the following equipment was recently published elsewhere [17]. The different bike types had a price that was manageable for most families with young children, representing a realistic alternative. Cycling was entirely voluntary, namely the intervention component was bike access only, without cycling directions. All participants in the intervention group (*n* = 18) were invited to participate and agreed to participate in focus group interviews after each intervention arm.

Thus, for this qualitative study, the 18 participants in the intervention group were the sample. The project was conducted according to the guidelines in the Declaration of Helsinki [23], and research clearance was assigned by the Norwegian Center for Research Data (number 52964). All participants were provided written information on the study’s objectives and methods before providing consent electronically. The trial was registered at clinicaltrials.gov with number NCT03131518, and the manuscript was checked for accordance with the COREQ 32-item checklist [24].

### Focus group interviews

Three focus group interviews were conducted shortly after each cycling period, that is, in December, March, and June, in total nine interviews. Such a longitudinal data collection, comprising different seasons and usage of different bike types, allows for greater variations and nuances in experiences. In Norway, substantial seasonal variations in cycling activity are normal because of the climate. Notably, the winter was atypical for southern Norway, that is, colder with more snow and ice, over a longer time, than normal. Additionally, the autumn was rainy, and the spring was late. Thus, cycling during the duration of this period was more challenging than usual. Each focus group interview included four to six participants (due to some non-attendance; Table 1) that had used the same type of bike for the previous three months, and the duration of all interviews was approximately 60 minutes. The interviews were held at 8.00 p.m. so that participants with young children could attend. The participants were served light refreshments, that is, coffee, mineral water, fruit and nuts, to create an informal, friendly environment.

**Table 1:** Participation in the nine focus group interviews.

Cycling period	Focus group number and bike type	Possible number of participants	Number of participants attending	Reason for non-attendance
**Autumn**	1: E-bike	6	4	Work (*n* = 1), illness (*n* = 1)
**(Sep–Nov)**	2: Longtail	6	5	Illness (*n* = 1)
	3: Traditional bike	6	5	Unknown (*n* = 1)
**Winter**	4: E-bike	6	5	Illness (*n* = 1)
**(Dec–Feb)**	5: Longtail	6	6	
	6: Traditional bike	6	5	Illness (*n* = 1)
**Spring**	7: Traditional bike	6	4	Work (*n* = 1), no babysitter (*n* = 1)
**(Mar–May)**	8: Longtail	6	5	Unknown (*n* = 1)
	9: E-bike	6	5	No babysitter (*n* = 1)

The participants’ seating arrangement was according to Krueger and Casey’s recommendations [25], that is, the most-active talkers were seated closest to the moderator, and the less-active talkers were placed more distant to the moderator, making eye contact and involvement in discussions easier. Further, a semistructured interview guide was developed according to a recommended focus group methodology [25], but with the exact wording formulated by the authors, to initiate the discussions. The interview guide (Table 2) comprised one opening question, one introduction question, eight key questions, and one ending question. The questions addressed participants’ experiences with and perceptions of cycling for transport, including facilitators of and barriers to cycling (i.e. motivation). Before the first focus group, the interview guide was pilot tested on four individuals from the target group (other than the participants).

**Table 2:** Semistructured discussion guide for the focus group interviews.

Question type	Purpose	Question	Timing (min)
**Opening**	*To get participants acquainted and feel connected*	First, I want all of you to present your name and the main reason for signing up for this research project.	5
**Introduction**	*To begin a discussion of a topic*	Now, I would like you to take two minutes writing down keywords for what, in your opinion, characterises a well-functioning mode of transport in general—it does not need to be a bike.	5
**Key**	*To obtain insights into the main subject of the study*	1: Can you describe one specific day when you cycled? Independent of positive or negative experiences, or a combination of both.	40
		2: How can you relate the keywords characterising a well- functioning mode of transport in general to your “cycling- days”—for instance, this specific day?	
		3: What made you cycle instead of using the car?	
		• were any factors more motivating than others?	
		4: If you recall one day when you ended up driving instead of cycling—can you describe this day?	
		• what would need to be different to make you cycle on this day?	
		5: Did you find any factors more limiting to your cycling than others?	
		6: How did you experience the (physical activity) intensity when cycling?	
		7: Can you tell me how the family as a whole has been affected by your participation in this project?	
		8: At this point, what do you think about cycling for transport after the trial period and onwards?	
**Ending**	*To bring closure to the discussion*, *including a recap from the assistant researcher*	Is there anything you would like to add that we have not discussed yet?	10

At the start of each focus group, the moderator (HBB: first author, female, age 34 years) informed the participants of the following: the purpose and procedures and that the discussion was audio recorded, their information would be confidential, and anonymous transcriptions would be used. Most of the allocated time was spent discussing key questions to explore how the participants experienced cycling for transport with the different bike types during the different seasons. The moderator used the interview guide to lead the discussion and allowed sufficient time for participants to answer questions and respond to comments from other participants while keeping the discussion on track and the participants involved. The assistant moderator (TW: second author, male, age 41 years) assisted the discussion by observing, taking notes, and asking the participants questions to clarify concerns (twice during the discussion and once at the end of the discussion).

Immediately after each focus group, the moderator and the assistant moderator, who both held a PhD in health science and worked in public health, evaluated the discussion by sharing first impressions and assessing the role of the moderator. The moderator had worked quantitatively, and the assistant moderator had experience with both quantitative and qualitative research methods, including focus group interviews. Both moderators were involved in all parts of the data collection in the research project, of which the present qualitative study was one part. Both moderators had an active lifestyle, including active transport, and on several occasions during the trial period, they met the participants by coincidence while being physically active.

### Data management and analysis

Audio recordings of the qualitative interviews were transcribed verbatim in Norwegian. Next, data were imported into the software analysis programme NVivo V.11 (QSR International), and a systematic text condensation was conducted by HBB, with assistance from TW. In addition, three of the co-authors (LF, EB, and SB) were involved in the analysis: They discussed coding, subthemes, and main themes. The procedure of Malterud [26] was followed and entailed a four-step inductive approach. First, the text was read as a whole to obtain an overall impression and to note tentative themes. Second, meaning units (i.e. the words, sentences, or paragraphs containing aspects related to each other through their content and context) were identified, before being condensed and coded close to the participants’ descriptions. Third, the condensed meaning units were regrouped into subthemes by pooling data with similar characteristics together in a theme defined by its content. Fourth, the subthemes were synthesised into main themes (Table 3). Translations from Norwegian to English were performed for quotations reported in the manuscript and conducted by HBB in collaboration with a native English-speaking language editor.

**Table 3:** Illustration of the relation between meaning units, condensed meaning units, subthemes, and main themes from systematic text condensation.

Meaning unit (quotation)	Condensed meaning unit/code	Sub-theme	Main theme
When you have not cycled and you are not used to it, there’s pretty much new things to familiarise yourself with. To prepare the day before, you know, before you felt that it became a routine. Just…if it was raining one day, you just: “ok, I didn’t think of that”, you know.	When you are not used to cycling, there is a lot to familiarise yourself with before it becomes a routine.	The meaning of cycling skills and habit.	Cycling is cumbersome.
I’ve kind of cycled a bit before I had kids, and I thought this [the project] might be a motivation to do so [cycle again]. My husband has to some extent cycled; so, it appeared unwise to me if especially my daughter saw that her mother didn’t cycle, but her father did. So, I thought I should start cycling.	I thought this project might be a motivation to take up cycling again. It appeared unwise to me that my daughter saw her father cycle but not her mother.	What’s in it for me?	Cycling reflects the desirable me.
I’ve learned that it’s possible. Even if you are delivering two kids to kindergarten, it’s possible to cycle. Because I hadn’t tried that before this project.	It’s possible to cycle, even if you are delivering two kids to the kindergarten.	Cycling is possible and feasible.	Breaking the cycling code.

## Results

### Participant characteristics

The intervention group comprised nine females and nine males, with mean age 35.8 years, ranging from 27 to 44 years. Sixteen (89%) participants were native Norwegians, and ten (56%) participants reported a high educational level (≥4 years of college/university education). Mean distances from home to the workplace, kindergarten, and grocery store were 6.9, 2.1, and 1.8 kilometres, respectively, and distances ranged from 1.5 to 14.1, 0.5 to 10.0, and 0.3 to 7.4 kilometres, respectively.

### Motivations for participation and characteristics of a well-functioning means of transport

Participants’ motivations for signing up for the project were diverse; however, individual factors such as own health, physical fitness, and being a positive role model for their children were more frequently mentioned than factors such as considerations for the environment and sustainability. Concerning characteristics of a well-functioning means of transport, the most frequently mentioned characteristics were ‘safe’, ‘efficient’, ‘comfortable’, ‘easy to use’, ‘sufficient cargo capacity’, ‘reliable’, ‘accessible’, ‘functional’, ‘flexible’, ‘fast’, ‘predictable’, ‘inexpensive’, ‘easy to park’, ‘child-friendly’, and ‘environmentally friendly’.

### Subthemes and main themes

#### Cycling is cumbersome

The first main theme, ‘cycling is cumbersome’, was synthesised from the following three subthemes: ‘it takes more planning and more time to cycle’, ‘the importance of weather and distance for cycling’, and ‘the meaning of cycling skills and habit’. The participants expressed that more planning and more time was necessary for cycling than for driving. They elaborated on considerations and remembrance of practical concerns when cycling, entailing a great deal of logistics and organising, which added to an already busy schedule and time scarcity:

…sometimes I had to both pick up and deliver, and you should be at work, and preferably he [the two-year-old son] shouldn’t have a very long day in the kindergarten, you know, so things should go rather fast. The overall needs to be dressed, and this and that, trailer and stroller that we need to take home on Friday and back to the kindergarten on Monday. Big trailer and so on, so there’s lots of hassle to get it all done… (father of one child, age 36 years, traditional bike during the winter season)

Two notable factors were identified concerning what was practically manageable: participants’ daily agendas and the need to combine several activities in limited amounts of time. For many, the car was preferred over the bike when work locations were farther away (e.g. if attending a meeting during the workday), when children had to be dropped off or picked up for various activities shortly after work, or when several tasks and destinations had to be combined. One of the fathers reported that the reason for using the car and not the bike, on non-cycling days, was usually due to such practical logistics:

It was something practical, that you should buy something of considerable size. Or find time for three to four things at once in the afternoon, or something like that. Or because of work. If, after working a long shift, and it just, you saw in advance that it just wouldn’t work. (father of two children, age 36 years, traditional bike during the spring season)

Participants also described how weather and road conditions, and the distance to the different destinations, influenced cycling. During the trial period, the autumn was challenging because of the many days with rain. One female participant described cycling on one rainy autumn day:

…I dressed myself and her [the four-year-old daughter], and started cycling, and it hadn’t become very chilly. So, I didn’t wear that much clothes, and I arrived at work soaked, and she was soaked. And the mascara was smeared out all the way back to the neck, and I just felt like “you know what: This I cannot bear!”. That’s a memory, and everybody at work was like “ok, so you are cycling today! That was the right day to choose! (mother of two children, age 38 years, e-bike during the autumn season)

The winter months were even more demanding because most of the period was characterised by snow, slippery roads, and sometimes, delayed ploughing of bike lanes. Additionally, the early part of spring entailed winter conditions, and one mother reported a difficult ride on the longtail in March, despite being equipped with winter tyres with studs, because of the combination of a long-lasting winter and an unfamiliar bike type:

I had never cycled on such a bike [the longtail] before—it was so heavy, and I struggled and I spun, and I had to walk with that bike. It was so heavy to walk with, I used like an hour home… The sweat ran, up and up those hills—I nearly died. It was completely impossible to ride in snow. (mother of two children, age 31 years, longtail during the spring season)

The experience of cycling being more cumbersome than driving relates to cycling skills and cycling habits. Because one inclusion criterion was not cycling to the workplace, kindergarten, and grocery store more than once per week in the 12 months before project’s start, these traits were not sufficiently present for many of the participants, especially not the cycling habit:

…when you have not cycled and you are not used to it, there’s pretty much many new things to familiarise yourself with. To prepare the day before, you know, before you felt that it became a routine. Just…if it was raining one day, you just: “ok, I didn’t think of that”, you know. (mother of two children, age 32 years, longtail during the winter season)

Nevertheless, great individual differences were observed concerning previous cycling experience and further cycling skills because some participants had cycled frequently (but not in the last year), commonly before they became parents. For those participants less familiar with cycling in general, the longtail was observed to be especially challenging, not least during the winter, because the longtail differs the most from a traditional bike because it is both longer and heavier:

It’s about skills. I learned to cycle when I was…first grade. And I have barely cycled before….so I think—I have never cycled with tyres with studs—so it felt insecure in a way, and when you are transporting two children as well, and one of them….is so little and cannot talk, you know…the longtail didn’t work at all for me—I found it uncomfortable, if I somehow fell, and…yes, I thought he [the one-and-a-half-year-old son] was vulnerable on that bike. (mother of two children, age 32 years, longtail during the winter season)

Although increased time use and need for more planning were mentioned for all three bike types, several participants experienced that the e-bike made cycling less cumbersome because of the ‘ease’ provided by the engine. One of the participants described a feeling of effortlessness on a physical, mental, and practical level:

The cycling experience becomes a bit different when you just fly away. One thing is that it became easier to cycle, but there is also an ease on the mental level. Suddenly there’s no strive. I’m often kind of hungry on my way home from work, and now that wasn’t an issue either. With the previous bike [tested within the project] I often had to stop at the gas station to buy a chocolate before I continued cycling. (father of three children, age 44 years, e-bike during the winter season)

Other participants stressed the reduced exercise effect due to the assistance provided by the e-bike; hence, their opinion of the e-bike was less positive compared with a traditional bike. In addition, many participants experienced an increased risk of technical problems when riding an e-bike, including a discharged battery—especially during the winter season, because battery range is limited in low temperatures. Additionally, because e-bikes are much heavier than regular bikes, cycling them without assistance was strenuous:

One learns two things: To check the weather forecast and to charge the battery. Because I had one trip…I have two children in the trailer, one four-and-a-half-year-old and one just over a year, and together they weigh more than 30 kilos. So, it’s heavy, and the battery-sign [on the display] starts blinking when you have no time for charging, and there’s no backup or anything like that. And it’s strenuous to cycle an e-bike too [without the electric assistance]. (mother of two children, age 32 years, e-bike during the autumn season)

#### Cycling reflects the desirable me

The second main theme, ‘cycling reflects the desirable me’, was synthesised from the two subthemes ‘what’s in it for me’ and ‘other’s perceptions of me and my transport habits’. Frequently mentioned facilitators were personal factors such as exercise and physical activity, participants’ health, and being a positive role model for their children:

…I’ve kind of cycled a bit before I had kids, and I thought this [the project] might be a motivation to do so [cycle again]. My husband has to some extent cycled, so it appeared unwise to me if especially my daughter saw that her mother didn’t cycle, but her father did. So, I thought I should start cycling. (mother of two children, age 43 years, longtail during the autumn season)

Additionally, logistics and comfort were observed to be more important than environmental sustainability for most participants, and a continual negotiation with themselves regarding cycling was described because the ‘default’ mode for everyday travel was a car for most participants. A conflict between convenience and the will to cycle was described:

…my motivation was to move more. But the lazy part of me wants to move less. So, it’s a fight; you’re supposed to get in shape and move more, yet sometimes the laziness wins. And the time-thing. It’s easy to blame for not taking the bike. (father of two children, age 34 years, e-bike during the autumn season)

The participants reported different responses and feedback from others, and the extent of social support from family, friends, and colleagues clearly affected transport habits. Using a bike for everyday travel was recognised as positive. However, the type of bike obviously mattered, and many work colleagues and members of the public considered the e-bike cheating, resulting in conflicting feelings for some participants. On the one hand, there was the positive experience of ease, and on the other hand, there was the perceived social stigma attached to e-biking. Based on the participants’ experiences, we differentiated three groups:

- participants who preferred the e-bike publicly:
  Yes, I’m very pleased. Compared to the two other bikes, I think it was so much easier to cycle with the kids, and it was actually faster than driving. Once we drove, I saw the traffic jam, and I just turned around and we took the bike instead. It goes equally fast, or faster. (mother of three children, age 32 years, e-bike during the spring season)
- participants who preferred the e-bike but found it embarrassing because of perceived social stigma:
  …and when I passed, and the engine made noise, I was kind of ‘I hope no one sees me’. Sort of embarrassing! I thought: ‘now I’m lazy, I can actually cycle without assistance. (mother of two children, age 31 years, e-bike during the winter season)
- participants who preferred a traditional bike because physical activity and exercise was considered more important than the ease provided by the e-bike:
  …if we were having a car number two, I could seriously consider an e-bike, yet it doesn’t meet my intentions [of exercising]. So, for me it was easier to return it [the e-bike], because then I was forced back to a regular bike, and now I’m kind of on with it again. (father of three children, age 39 years, e-bike during the spring season)

The longtail received a great deal of attention from others, both children and adults, and was a bike type that most of the children really liked. The children thought the longtail was cool, they were closer to mom or dad as they were sitting on the same bike, and were closer to the surroundings, compared with sitting in a trailer. One father described the children’s fascination with the longtail:

When we cycled around, everyone wanted to sit on the bike; I had to take like ten-fifteen kids for a round and just take turns. ‘No, now it’s my turn. I want to…’ So, everyone wanted to sit on the bike; they thought it was really cool. (father of three children, age 31 years, longtail during the autumn season)

Nonetheless, the participants’ experiences with the longtail revealed a diversity between two extremes, which seemed to be highly gender related. For some participants, the longtail worked out fine, that is, it was comfortable, easy to ride considering the size, and had plenty of space for cargo (yet was a bit heavy):

I found that bike nothing but positive. We get up, go to the kindergarten to deliver, then to work, and back home again. And I’ve been to the grocery store shopping, I carried six shopping bags on that bike, and thought it was great. Bottles of soda and everything—it was heavy. And we went for a sled ride, carrying two sleds on it…enjoyable. And junior [the four-year-old son] think it’s great fun to ride that bike, so that’s nothing but positive. The only drawback is the weight, it’s heavy. You become sweaty, no matter where you go. (father of one child, age 37 years, longtail during the winter season)

For other participants, the longtail did not work out at all, that is, it was as too big and heavy, causing a feeling of insecurity and lack of control:

I thought, on my one trip from here, when I cycled alone, that it was ok. But once I picked up—I have one almost three-and-a-half-year-old—and when I picked her up, there’s really just one steep hill up and one steep hill down, and I thought it was really uncomfortable—so I just had to stop. I just had to walk with her, and even when walking with the bike I felt having little control. (mother of two children, age 37 years, longtail during the winter season)

#### Breaking the cycling code

The third main theme, ‘breaking the cycling code’, was synthesised from the four subthemes: ‘cycling is feasible’, ‘the children’s attitude influences my cycling’, ‘a normalising of cycling in the family’, and ‘freedom of cycling’. Although the participants indicated that cycling was generally more demanding than driving a car, many found cycling for transport feasible, including in winter, for dropping off or picking up one or more children at kindergarten:

I’ve learned that it’s possible. Even if you are delivering two kids to the kindergarten, it’s possible to cycle. Because I hadn’t tried that before this project. (mother of two children, age 43 years, e-bike during the spring season)

And the worst days I’ve cycled—I was really worried about picking up the kids, because I expected it to become bad…But it turned out positive, the youngest one [the three-year-old son] sat singing. So, I was just like “wow”. This works out, it’s just about dressing… (mother of three children, age 32 years, longtail during the winter season)

Some participants even reported that cycling became easier than driving after a while:

I thought it was very comfortable with a bike. I sort of lost more and more that the car was comfortable, I would rather cycle…Ultimately, it wasn’t more hocus-pocus [i.e. difficult] with the bike than with the car. (father of one child, age 37 years, traditional bike during the spring season)

One male participant described the first weeks of the trial, that is, how cycling was exhausting but became enjoyable:

…it actually went quite fast as well. From being nothing but painful to being fun. And then you started catching up to the seventy-year-olds [other cyclists, being older], and then you start to get annoyed over the e-bike users, because they cheat. And then you start catching up on the others [other cyclists]. (father of three children, age 39 years, traditional bike during the autumn season)

The participants who broke the cycling code and became less car-dependent had common factors that contributed to their success; among them, what was observed to be crucial was cycling until it became a habit:

Now, I think that I cycle to work–that’s what I do. It’s kind of nothing I choose to do, it’s something I just do, in a way. (father of three children, age 44 years, longtail during the spring season)

Also relevant was a basic attitude entailing that the pros of cycling were greater than the cons, which was emphasised by one female participant as follows:

It’s mainly that the positive outweighs the negative. Yes, sometimes there’s some time pressure. But you don’t need to work out at the gym. At least for me, not having the opportunity to do so. The cycling is the exercise I get. Plus, the kids [the two-year-old and the four-year-old sons] love to cycle. And yes, it will be slippery, and it will be cold, but it’s worth it. So, for me, it may seem like major barriers, but in the long term, it’s not. (mother of two children, age 27 years, traditional bike during the autumn season)

However, planning and conscious choices were still required for cycling to be conducted. One of the mothers reported that she had to make an appointment with herself, the day before, that she would cycle to work the next day, regardless of the weather:

..to decide that at this and that point I shall cycle, no matter what the weather is like, and almost no matter what the road conditions are like. And in a way planned it and made almost an appointment with myself, before going to bed. (mother of three children, age 39 years, e-bike during the spring season)

In addition, support from work colleagues and from family was of great importance to this mother, to get her to cycle. Additionally, feeling that her physical fitness improved was a key motivator. In summary, we observed that it was much more about overcoming barriers and thereby experiencing mastery:

And then have supporters at work—I did so. That’s a motivator for me. And of course, there’s something about the physical fitness; I felt that I should have conducted the test [cardiorespiratory fitness] after the longtail. Because then I felt in much greater shape than what I might be now [after using the e-bike]. But…if I just get over the barrier and see that it works, that’s also a motivator for me. (mother of three children, age 39 years, e-bike during the spring season)

Related to this phenomenon, the participants expressed the importance of incorporating a routine for cycling for the transition from the car to the bike being the default transport mode. After a while, some participants experienced the physical and mental advantages of cycling, such as the joy of moving and improved wellness from being outside in the fresh air:

…but I remember from this winter, that even when the weather was bad, the colleagues just said, ‘did you cycle today?’ and then they laughed. But it was never that bad when you got out. And it gives kind of joy to move, to have some fresh air and…well, you simply feel better. (father of three children, age 39 years, e-bike during the spring season)

However, for most of the participants, the greatest facilitator for breaking the cycling code was the children and their attitude toward cycling, that is, whether the children wanted to cycle or not. Nearly all the participants experienced that their children enjoyed cycling:

I was very surprised how fun she [the four-year-old daughter] thought it was. I thought we would have to work on it, but she was more like, “What, mom aren’t we going to cycle soon?” So, she thought it was great fun… (mother of two children, age 38 years, e-bike during the autumn season)

Many participants described that the children usually preferred cycling over driving, and several participants’ children requested cycling. The children even exerted extra effort to encourage their mom or dad to cycle for transport instead of driving:

And I experienced that the four-year-old thought it was great fun to cycle, so she asked, or she said that she wanted to cycle. And some days we were a bit late, and then I said, “Then you’ll have to be quicker the next day”. And that helped, then she was quicker. So, she sat singing on the bike. (father of two children, age 36 years, longtail during the autumn season)

For one mother, cycling was necessary to keep the peace at home:

The boys [the two-year-old and four-year-old sons] love to cycle, it’s harder not to cycle than to cycle. Because they get really angry if I don’t cycle with them. They motivate me more than I motivate myself. So, if I want to take that half hour of arguing when I say we shall walk and not cycle, or take the car… They don’t want to. They want to cycle. (mother of two children, age 27, traditional bike during the autumn season)

An exception was one father for whom all went wrong when he cycled when his two-and-a-half-year-old son did not want to cycle. The son had a tantrum and was obstinate the rest of the day, causing a mental anguish for his father. However, this episode was likely related to the son’s age, rather than cycling, and was the only negative episode reported. All the other children were observed to enjoy cycling and preferred cycling over driving.

Moreover, ‘normalising’ of cycling in the family was discussed after the project. In other words, cycling for transport was to a greater extent than earlier considered a realistic alternative to driving a car for everyday transport and leisure purposes, for example, cycling trips:

… it has somehow struck me–the twelve-year-old, who is like the “car-generation” being driven around, said “but I can just cycle, mom”. Yes, she kind of said that, such a comment… (mother of three children, age 39 years, e-bike during the spring season)

…what I notice happening is that it’s more normalised, that it’s more like a daily “misdemeanour”, and that’s what the children’s mom says as well…they typically ask, “Can we go for a cycling trip?”. And then she prepares the child seat and all this, and they go cycling…So it’s kind of dragged into everyday life, like a normal alternative to the car… (father of three children, age 39 years, e-bike during the spring season)

Notably, without mentioning the term, one participant described what ‘freedom of cycling’ could entail in practice; that is, compared with driving a car or using public transport for everyday travel, cycling could be flexible, accessible, and predictable:

…the travel to work starts long before leaving the house. And that’s where flexibility and time comes in. We have three kids, and from the time I wake them up, so begins a race against the clock….they have to get ready, have to eat, have to dress…and one of these things that I like about cycling, is that…ok, if it fails here, it’s only me being delayed, but it doesn’t multiply. If I’m two minutes late at the bus stop, it doesn’t mean 12 minutes of waiting. Like it can mean when taking the bus. Or when being stuck in a traffic jam. (father of three children, age 44 years, traditional bike during the autumn season)

Unfortunately, some participants had insufficient motivation to replace their car with a bike for everyday travel, independent of weather conditions. One father said that he was not there yet:

But I do see people cycling the whole year, and it’s possible. But I don’t know what it takes to motivate me to…even if the wind blows sideways, to get on the bike—I’m not quite there, yet at least. (father of one child, age 36 years, traditional bike during the spring season)

However, also for the participants not breaking the cycling code, that is, cycling for transport was perceived as infeasible, the project was observed to affect the family to some extent. The participants reported being inspired to cycle more in general, for exercise or recreation, and an increased consciousness of cycling. One mother reported on her husband: He suddenly joined her and their two sons on cycling trips and wanted to buy a new bike:

My husband wants a new bike, so it would be wrong saying that he’s not affected. Because he did try it. And we have cycled all four together. He used to hate cycling: “it hurts to sit on, and this and that”. But now we have cycled together, the four of us, so something clearly happened. (mother of two children, age 31 years, e-bike during the winter season)

Last, one female participant who did break the cycling code and experienced a transition away from car-dependency summarised her main outcome from the project as follows:

…so this project made me go from being 100% car driver to about 70% cyclist; I guess, yes. …I’m not cycling every day, but I have to like three days a week…and I don’t have to in November, December, and January. But I aim to cycle the rest of the time. Yes, that’s thanks to you [the project]. (mother of three children, age 39 years, e-bike during the spring season)

## Discussion

The participants’ descriptions revealed that many participants perceived that cycling for transport was feasible, including in winter, but others found everyday cycling challenging. Several participants reported the e-bike was feasible due to the ‘ease’ of electric assistance. Additionally, the e-bike was frequently reported to fulfil many of the relevant characteristics of a well-functioning means of transport. By contrast, some participants emphasised that they experienced a decreased exercise effect compared with a traditional bike, which reduced their perceived advantages of the e-bike. The human-powered longtail was observed to be a great option for everyday travel or did not work, mainly due to the larger size and weight. Three main themes summarise the participants’ experiences: ‘cycling is cumbersome’, ‘cycling reflects the desirable me’, and ‘breaking the cycling code’.

### Cycling is cumbersome

Regarding the experience of cycling as cumbersome, more planning for cycling compared with driving and, for some, increased time use were relevant factors. Notably, time use seemed to depend on the distance to the destinations and time of the day, that is, degree of congestion. For participants who lived closer to their workplace, and in more densely populated areas, cycling was equally fast or faster than a car, especially when riding the e-bike and during rush hour. Correspondingly, in a small sample of novice e-bike users in Canada, the participants emphasised that when accounting for the time necessary for parking, the total commute time for an e-bike and a car was comparable [27], and in a Dutch study, the duration of commutes by e-bike was approximately as long as bus commutes but longer than car commutes [18]. Additionally, in a study in the San Francisco Bay Area [15], participants and cargo e-bike users emphasised that compared with driving a car during rush hour, the commute by bike was far more time reliable.

Regarding additional planning, this factor may be related to the convenience and ease of driving a car, combined with no cycling habit. When unfamiliar with cycling, organising and logistics could cause additional hassles until a cycling routine is established. However, some daily agendas and tasks would probably not be facilitated simply by planning and developing a routine, for example, when having to combine several tasks and destinations within a relatively short time, or when buying things of considerable size. In such cases, a car was often considered the only manageable transport option in our sample, even when considering the e-bike (in addition to the traditional bike and the longtail). The finding of lower bike use for journeys with a greater number of destinations agrees with Dutch data on e-bike users [18].

Most of our participants reported that road conditions, temperature, and weather limited cycling. Similarly, Plazier and colleagues [18] demonstrate that for most of the e-bike users, rain was a major influence, which entailed careful planning. Similar to the participants in our study, weather apps were checked the night before, and rain clothing was eventually prepared. However, an even greater impact factor for our participants was the winter because this season entailed snowy, icy roads and freezing temperatures for nearly three months. The e-bike had pros and cons in this regard. The power provided by the engine, together with weight and grip, could make it easier to navigate through snow, but mainly for the more avid cyclists. This finding was supported by Edge et al. [27], that is, the more enthusiastic cyclists were more likely to ride their e-bike in all weather conditions and through all seasons. In addition, battery range was markedly reduced in lower temperatures, which has also been reported in other studies [27, 28].

We observed that participants’ cycling skills differed and depended on previous cycling involvement. For participants unfamiliar with cycling, cycling in winter, especially with the longtail, was demanding. Some participants, mainly the mothers, reported feeling insecure and without control because of the size and weight of the longtail; additionally, because of their low confidence, they were less inclined to use the longtail. Similarly, US mothers riding cargo bikes emphasised the importance of bike education to be a confident cyclist who feels safe and secure on a cargo bike [29]. However, when TW suggested that cycling education or training might increase the confidence in riding the longtail, the participants did not approve of it. Many participants were observed to have a general attitude that riding a bike is easy; thus, cycling education was redundant.

### Cycling reflects the desirable me

The second main theme derived from the participants’ experiences, that is, ‘cycling reflects the desirable me’, embraces both an individual perspective and a more social perspective. Parents’ frequently mentioned motives for increasing everyday cycling were exercise and PA, their health, and being a positive role model for their children. Additionally, logistics and comfort were observed to be more important drivers than environmental sustainability for most participants. Some participants had cycled earlier, either for transport or exercise, typically before they became parents, and wanted to start cycling again to increase their PA and physical fitness. For participants who valued physical activity and exercise over the ease provided by the e-bike, the traditional bike was the preferred bike type. Similarly, a Norwegian study found that a large proportion of the cycling population reported that improved fitness was the main motivation for cycling [30]; thus, the e-bike was counter to their main motivation. Further, our participants recognised the importance of showing their children the potential of the bike to replace the car as a transport mode because this could affect the children’s travel habits. Similar to participants in this study, Plazier and colleagues [18] report that the main motive of e-bike adoption is health rather than the environment, whereas Edge et al. [27] demonstrate that the environmental benefits of reduced car use, a more physically active commute, and the testing of new technology were relevant drivers for trial participation, depending on the mode of transport being replaced.

The social perspective and the response and feedback from others, that is, family, friends, work colleagues, and members of the public, affected travel habits. In general, cycling for transport was recognised as positive, and bike type was a deciding factor. For participants concerned with others’ opinions, the ease of the motorised assistance provided by the e-bike conflicted with the perceived social stigma, that is, e-bike use was considered ‘cheating’. Notably, e-bike use was demonstrated to entail MVPA [12]. The social stigma associated with e-biking is also identified by Jones et al. [28] and Thomas [15] in e-bike users in the United Kingdom and the Netherlands, respectively, and in cargo e-bike users in San Francisco; however, these e-bike users were not deterred by the perceived social stigma. Nonetheless, individuals reluctant to deviate from mobility norms could be discouraged from using an e-bike when experiencing social stigma. By contrast, the longtail received plenty of positive attention from others, both children and adults. Other parents wanted to know about the project, what type of bike the longtail was, and how it could be used, that is, a completely different interest what was experienced when riding the e-bike. Additionally, the longtail seemed to be liked by among most children. Likely, the greater interest in the longtail was because this bike type is unusual in southern Norway; thus, many onlookers had neither attempted to use nor seen it before.

### Breaking the cycling code

The last main theme was ‘breaking the cycling code’. Many participants cycled significantly more and became less car dependent because year-round cycling was feasible for them. For the participants ‘breaking the cycling code’, the bike replaced the car or public transport, namely motorised transport. The literature [17] has demonstrated that seven participants managed to switch from a car to a bike as their main transport mode (to the workplace) during the trial period, and were thus classified as cyclists at follow-up. In addition to these seven participants, some became less car dependent, namely they cycled more and drove less than what they did before participating in project CARTOBIKE. One father even said that cycling felt easier than driving—after a while. For the participants who succeeded in breaking the cycling code, they expressed that some factors were more relevant than others, such as deciding to cycle, planning and conscious choices, focusing on the pros of cycling instead of the cons, acquiring social support, and incorporating a cycling routine. However, the most important facilitator, emphasised by all participants, was the children and their attitude towards cycling. With one exception, all the participants’ children enjoyed cycling and requested cycling over driving. The parents also described their appreciation for the different levels of interaction they had with their children when using the longtail, compared with using the trailer for the e-bike and the traditional bike, and not least compared with driving a car. These findings corroborate Thomas [15], who investigates a sample of parents using cargo e-bikes for family transport in the San Francisco Bay Area and demonstrates that becoming a parent does not have to end cycling for transport.

An additional aspect mentioned by several participants was a ‘normalising’ of cycling in the family after the trial. That is, the trial generated an attitude that the bike could function as a realistic transport mode and an alternative to the car for everyday travel and leisure, such as cycling trips. Furthermore, participants who did not break the cycling code and found cycling infeasible reported a change in their family after the project. This finding may have occurred because of the increased consciousness of cycling or an inspiration to cycle more. Additionally, ‘freedom of cycling’ was described without being mentioned explicitly, that is, the flexibility, predictability, and independence of cycling. Similarly, freedom, or independence from public transit schedules or carpooling, has been highlighted as the main motive for e-bike use for everyday travel [18]. Likewise, US data demonstrated that the greater mobility and freedom of travel provided by the cargo e-bike were highly valued [15]. In addition, ‘freedom of cycling’ could include the feeling of well-being related to being physically active outdoors, which was mentioned as a relevant motivator by many participants. In addition, participants expressed that the commute by bike created time to mentally prepare for the day ahead or to disconnect from work on the trip home. Such intrinsic utility from cycling was described among e-bike users in the Netherlands [18] and could be connected to the newly proposed four components that explain why cyclists are consistently the happiest commuters [16].

### Strengths and limitations

According to our review of the literature, this study is the first to explore the perspectives of parents with children attending kindergarten on the use of different types of bikes for year-round, everyday travel to the workplace, kindergarten, and grocery store. The participants were interviewed after exposure to each bike type for three months; thus, the foundation for their post-intervention reports should have been sufficient. Hence, this study contributes to the literature on the feasibility of cycling for transport for busy parents of young children and increases the understanding of the barriers to and motivations for the use of sustainable transport when travelling with young children [5]. Notably, the cultural, geographical, and infrastructural context in this study differs significantly from those in typical cycling cultures, such as in the Netherlands and Denmark, because Norway is a car-dominated country with low temperatures and snowy and icy winters, making year-round cycling more demanding.

The focus group interviews provided in-depth knowledge of parents’ experiences with cycling for transport using different bike types that enabled an evaluation of the feasibility of the CARTOBIKE intervention [22] and elaboration on its quantitative findings [17]. Additionally, including all three bike types over three seasons provided greater variation and nuances to the focus group findings than was found in those of the literature. Focus groups of four to six participants was suitable for the parents to express their experiences and to facilitate a dialogue between the parents during the interviews, providing richer data than what would be obtained through individual interviews. Although we were limited to participating parents, the moderators’ posited that saturation was reached, that is, additional themes and understanding would not emerge if additional parents were included. Further, because both moderators had an active lifestyle, including active transport, they were familiar with the topic of interest, which may have been a strength of this study. However, this personal interest and engagement could have affected the direction of the interviews, for example, follow-up questions and elaborating questions. Nonetheless, the systematic approach applied to the data analysis could reduce the limitations related to subjectivity.

However, using focus groups for data collection could have weaknesses, depending on the group dynamics [31]. If a dominant individual makes a statement, others may hesitate to contradict this statement. Even if some participants were more extroverted and active than others, maybe those seated closest to the moderator [25], such patterns were not observed. Further, qualitative research is interpretative and may not be generalisable. Nevertheless, the transferability of findings to similar conditions and populations may be possible if the cultural, geographical, and infrastructural contexts; previous cycling experiences; and sociodemographics are considered.

### Implications

The CARTOBIKE intervention [22], entailing bike access as the only intervention component, was recently found to positively influence cycling amount, transport habits, and intrinsic motivation for cycling [17], consistent with the socioecological framework [32] and suggesting that accessibility is a relevant environmental determinant for PA, including cycling for transport [33]. In addition to these quantitative findings, the participants’ experiences revealed that cycling with young children in Norway entails several challenges; however, despite hilly terrain and cold, snowy winters, many reported that cycling was feasible and enjoyable. Changing one environmental determinant, that is, bike access, facilitated novel and positive experiences related to cycling as well. Similarly, direct experience as a behavioural change technique was shown to have a positive influence on attitude, beliefs, and in case of a positive experience, self-efficacy [34]. In addition, for most of the participants in this study, planning was a concern, which fits behaviour change theories and the use of self-management skills [35].

In summary, this understanding could inform interventions aiming to increase everyday cycling in parents of young children and the work of practitioners targeting cycling promotion in this group. Further interventions providing bike access, and thereby direct exposure, could be an advantage over fulfilling individual preferences for bike type to a greater extent and might be caused by addressing the process of self-management. Thus, cycling would probably be experienced as more feasible by more parents and have further potential to increase cycling. In addition, some form of cycling education, for example, relevant tips and tricks, may make novice cyclists feel more confident and safer on a bike, which could also facilitate cycling. Furthermore, increasing parents’ awareness that most children seem to love cycling and prefer cycling over driving may persuade parents to ride a bike rather than drive a car. Finally, considering the promising findings on cargo e-bikes as an alternative mode choice for family transport [14, 15], combined with experiences from this study, electric longtails would probably provide a favourable combination of the carrying capacity of a longtail yet with the ease of an e-bike, as well as fun for both parents and children. Hence, the potential of electric longtails for everyday travel in parents of young children should be further explored in larger studies. Notably, electric longtails are expensive but economically viable if replacing a car.

### Conclusion

Findings in this study indicate that, despite challenging weather conditions, parents of young children may experience cycling as cumbersome but desirable and that bike access could contribute to increasing the feasibility of cycling every day.

## Supporting information

S1 Fig Bike types and trailer. The bike types and the trailer used in project From Cars to Bikes.
(TIF)

## Abbreviations

MVPA: moderate-to-vigorous intensity physical activity
PA: physical activity
E-bike: Electric-assisted bicycle

## References

[1] Trost SG, Owen N, Bauman AE, Sallis JF and Brown W. Correlates of adults' participation in physical activity: review and update. Med Sci Sports Exerc. 2002; 34: 1996–2001. 12471307 10.1097/00005768-200212000-00020

[2] Bauman AE, Reis RS, Sallis JF, Wells JC, Loos RJF and Martin BW. Correlates of physical activity: why are some people physically active and others not? Lancet. 2012; 380: 258–71. 22818938 10.1016/S0140-6736(12)60735-1

[3] Sahlqvist S, Song Y and Ogilvie D. Is active travel associated with greater physical activity? The contribution of commuting and non-commuting active travel to total physical activity in adults. Prev Med. 2012; 55: 206–11. 22796629 10.1016/j.ypmed.2012.06.028 PMC3824070

[4] de Nazelle A, Nieuwenhuijsen MJ, Anto JM, et al Improving health through policies that promote active travel: a review of evidence to support integrated health impact assessment. Environ Int. 2011; 37: 766–77. 21419493 10.1016/j.envint.2011.02.003

[5] McCarthy L, Delbosc A, Currie G and Molloy A. Factors influencing travel mode choice among families with young children (aged 0–4): a review of the literature. Transport Rev. 2017: 1–15.

[6] Forsberg L. Managing time and childcare in dual-earner families: Unforeseen consequences of household strategies. Acta sociologica. 2009; 52: 162–75.

[7] Määttä K and Kyronlampi-Kylmanen T. Children's and parents' experiences on everyday life and the home/work balance in Finland. Int J Child Youth Family Stud. 2012; 3: 46–64.

[8] Rose J. “Never enough hours in the day”: Employed mothers’ perceptions of time pressure. Aust J Soc Issues. 2017; 52: 116–30.

[9] Jones RA, Hinkley T, Okely AD and Salmon J. Tracking physical activity and sedentary behavior in childhood: a systematic review. Am J Prev Med. 2013; 44: 651–8. 23683983 10.1016/j.amepre.2013.03.001

[10] Craigie AM, Lake AA, Kelly SA, Adamson AJ and Mathers JC. Tracking of obesity-related behaviours from childhood to adulthood: A systematic review. Maturitas. 2011; 70: 266–84. 21920682 10.1016/j.maturitas.2011.08.005

[11] Fishman E and Cherry C. E-bikes in the Mainstream: Reviewing a Decade of Research. Transport Rev. 2016; 36: 72–91.

[12] Bourne JE, Sauchelli S, Perry R, et al Health benefits of electrically-assisted cycling: a systematic review. Int J Behav Nutr Phys Act. 2018; 15: 116. 30463581 10.1186/s12966-018-0751-8 PMC6249962

[13] Shephard RJ. The Exercising Commuter: Is Commuting a Healthy Way to Be Active? Curr Cardiovasc Risk Rep. 2012; 6: 299–306.

[14] Schwartz J and Riggs W. PAPER No. 17–06482 The Impact of Cargo Bikes on the Travel Patterns of Women. *Transportation Research Board*,. Washington D.C Transportation Research Board 2017.

[15] Thomas A. A more sustainable minivan? an exploratory study of electric bicycle use by San Francisco Bay Area families. UC Davis: National Center for Sustainable Transportation 2016 https://escholarship.org/uc/item/6g79m3xx. Accessed 12 March 2019.

[16] Wild K and Woodward A. Why are cyclists the happiest commuters? Health, pleasure and the e-bike. J Transp Health. 2019; 14: 100569.

[17] Bjørnarå HB, Berntsen S, J te Velde S, et al From cars to bikes–The effect of an intervention providing access to different bike types: A randomized controlled trial. PLoS One. 2019; 14: e0219304. 31291314 10.1371/journal.pone.0219304 PMC6619759

[18] Plazier PA, Weitkamp G and van den Berg AE. “Cycling was never so easy!” An analysis of e-bike commuters' motives, travel behaviour and experiences using GPS-tracking and interviews. J Transp Geogr. 2017; 65: 25–34.

[19] Clifton KJ and Handy SL. Qualitative methods in travel behaviour research. *Transport survey quality and innovation*. Emerald Group Publishing Limited, 2003, p. 283–302.

[20] Morgan DL. *Focus groups as qualitative research*. Sage publications, 1996.

[21] Malterud K. *Fokusgrupper som forskningsmetode for medisin og helsefag*. Universitetsforlaget Oslo, Norway, 2012.

[22] Bjørnarå HB, Berntsen S, te Velde SJ, et al From cars to bikes–the feasibility and effect of using e-bikes, longtail bikes and traditional bikes for transportation among parents of children attending kindergarten: design of a randomized cross-over trial. BMC Public Health. 2017; 17: 981. 29282108 10.1186/s12889-017-4995-z PMC5745663

[23] World Medical Association. World medical association declaration of helsinki: Ethical principles for medical research involving human subjects. JAMA. 2013; 310: 2191–4. 24141714 10.1001/jama.2013.281053

[24] Tong A, Sainsbury P and Craig J. Consolidated criteria for reporting qualitative research (COREQ): a 32-item checklist for interviews and focus groups. Int J Qual Health C. 2007; 19: 349–57. 10.1093/intqhc/mzm042 17872937

[25] Krueger RA and Casey MA. *Focus groups*: *A practical guide for applied research*. Sage publications, 2014.

[26] Malterud K. Systematic text condensation: a strategy for qualitative analysis. Scand J Public Health. 2012; 40: 795–805. 23221918 10.1177/1403494812465030

[27] Edge S, Dean J, Cuomo M and Keshav S. Exploring e‐bikes as a mode of sustainable transport: A temporal qualitative study of the perspectives of a sample of novice riders in a Canadian city. The Canadian Geographer/Le Géographe canadien. 2018; 62: 384–97.

[28] Jones T, Harms L and Heinen E. Motives, perceptions and experiences of electric bicycle owners and implications for health, wellbeing and mobility. J Transp Geography. 2016; 53: 41–9.

[29] Riggs WW and Schwartz JE. The Impact of Cargo Bikes on Travel Patterns: Survey Report. 2015.

[30] Fyhri A, Heinen E, Fearnley N and Sundfør HB. A push to cycling-Exploring the e-bike's role in overcoming barriers to bicycle use with a survey and an intervention study. Int J Sustain Transp. 2017: 681–95.

[31] Nyumba T O., Wilson K, Derrick CJ and Mukherjee N. The use of focus group discussion methodology: Insights from two decades of application in conservation. Methods Ecol Evol. 2018; 9: 20–32.

[32] Sallis JF, Cervero RB, Ascher W, Henderson KA, Kraft MK and Kerr J. An ecological approach to creating active living communities. Annu Rev Public Health. 2006; 27: 297–322. 16533119 10.1146/annurev.publhealth.27.021405.102100

[33] Handy S, Van Wee B and Kroesen M. Promoting cycling for transport: research needs and challenges. Transport rev. 2014; 34: 4–24.

[34] Eldredge LKB, Markham CM, Ruiter RA, Fernández ME, Kok G and Parcel GS. *Planning health promotion programs*: *an intervention mapping approach*. John Wiley & Sons, 2016.

[35] Lorig KR and Holman HR. Self-management education: History, definition, outcomes, and mechanisms. Ann Behav Med. 2003; 26: 1–7. 12867348 10.1207/S15324796ABM2601_01

