# Supplementary figures and images for: Cumbersome but desirable—Breaking the code of everyday cycling
Source: PLoS One. 2020 Sep 14;15(9):e0239127. doi: 10.1371/journal.pone.0239127 (PMC7489513; doi:10.1371/journal.pone.0239127)

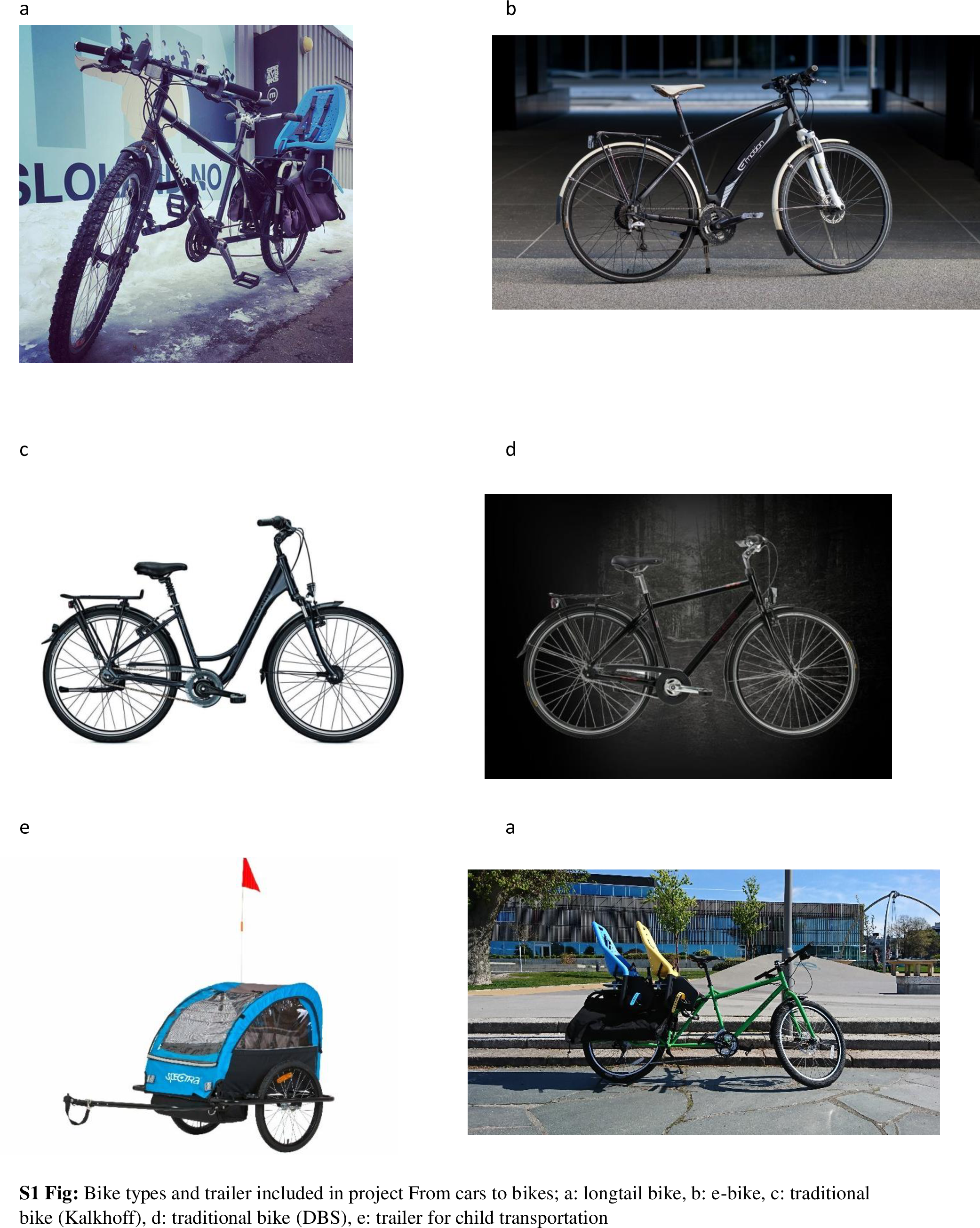

Supplement: S1 Fig — The bike types and the trailer used in project From Cars to Bikes. (TIF) [file pone.0239127.s001.tif]
